# Associations between Fitness Measures and Change of Direction Speeds with and without Occupational Loads in Female Police Officers

**DOI:** 10.3390/ijerph16111947

**Published:** 2019-06-01

**Authors:** Robin M. Orr, Filip Kukić, Aleksandar Čvorović, Nenad Koropanovski, Radivoje Janković, Jay Dawes, Robert Lockie

**Affiliations:** 1 Faculty of Health Sciences and Medicine, Bond Institute of Health and Sport, Bond University, Gold Coast QLD 4229, Australia; 2 Tactical Research Unit, Bond University, Gold Coast QLD 4229, Australia; 3 Police Sports Education Center, Abu Dhabi Police 253, UAE; filip.kukic@gmail.com (F.K.); cvorovic77@yahoo.com (A.Č.); 4 Specialized Physical Education at the department of Criminalistics, University of Criminal Investigation and Police Studies, 11080 Belgrade, Serbia; korpan82@gmail.com (N.K.); radejankovic@yahoo.com (R.J.); 5 Department of Helath and Human Performance, Oklahoma State University, Stillwater, OK 74074, USA; jdawes@uccs.edu; 6 Department of Kinesiology, California State Fullerton, Fullerton, CA 92831, USA; rlockie@Fullerton.edu

**Keywords:** law enforcement, foot pursuit, load carriage, chase, body armor

## Abstract

Female police officers may be required to pursue offenders on foot while wearing occupational loads. The aim of this study was to determine relationships between fitness measures and change of direction speed (CODS) in female police officers and the influence of their occupational loads. Retrospective data were provided for 27 female police officers (age = 32.19 ± 5.09 y, height = 162.78 ± 5.01 cm, and mass = 71.31 ± 13.42 kg) and included fitness measures of: lower-body power (standing long jump (SLJ)), upper-body and trunk muscle endurance (push-up (PU) and sit-up (SU)), aerobic power (estimated VO_2max_), and CODS (Illinois agility test). The CODS test was performed without and with occupational load (10 kg). Paired sample *t*-tests (between-load conditions) and Pearson’s correlations (relationships between measures) were performed with linear regression analysis used to account for the contribution of measures to unloaded and loaded CODS performance. CODS was significantly slower when loaded (unloaded = ~23.17 s, loaded = ~24.14 s, *p* < 0.001) with a strong, significant relationship between load conditions (*r* = 0.956, *p* < 0.001). Moderate to strong, significant relationships were found between all fitness measures ranging from estimated VO_2max_ (*r* = −0.448) to SU (*r* = −0.673) in the unloaded condition, with the strength of these relationships increasing in the loaded condition accounting for 61% to 67% of the variance, respectively. While unloaded agility test performance was strongly associated with loaded performance, female police officer CODS was significantly reduced when carrying occupational loads. A variety of fitness measures that influence officer CODS performances become increasingly important when occupational loads are carried.

## 1. Introduction

Police officers are required to carry out a variety of physical tasks that can range from attending a domestic disturbance and verifying a person’s identity [1] to chasing offenders on foot across varying distances [2,3]. Often these foot pursuits can occur while the officer is wearing their daily occupational load. Occupational loads for police officers can vary depending on job type, but they are typically around 10 kg for general duties officers [4] and over 20 kg [5], or even 40 kg [6], for specialist response police. For the general duties officer, these loads typically comprise essential equipment like a baton, radio, handcuffs, flashlight, etc. [4], and they often include body armor [7]. Specialist police loads are made heavier by the specialist equipment they must carry, which can include gas masks, riot or ballistic shields, and breaching equipment. Of note however, given that the average female officer is lighter than the average male officer [5], the relative load carried by female officers may be significantly heavier than those carried by male officers [4].

The impacts of occupational loads on mobility of the carrier are well reported in the literature, whereby measures of short-distance sprints [8,9,10], prone-start sprints [10,11,12], and agility runs [13] have been found to significantly decrease the mobility of tactically loaded participants. However, these studies were all conducted in military populations, who are known to generally carry heavier loads than law enforcement [4]. The findings of three studies on law enforcement that compared the impacts of body armor on police officer mobility were mixed with two studies [5,14] that found significant decreases in performance with added load and one study that did not [15]. Carlton et al. [5] found a significant decrease in time to complete an 80 kg dummy drag task when specialist tactical officers were loaded with 22 kg as opposed to their unloaded condition. Similarly, a study by Dempsey et al. [14] found that participants wearing stab-resistant body armor (7.65 ± 0.73 kg) significantly increased time to complete a simulated vehicle exit and sprint (mean time = 1.95 s loaded, 1.67 s unloaded, *p* < 0.001) and time to complete a mobility battery (mean time = 18.16 s loaded, 15.85 s unloaded, *p* < 0.001). Conversely, research by Schram et al. [15] found that there were no significant differences in completion time for the Illinois agility run performance between officers wearing duty loads, which included body armor (10.8–11.5 kg) when compared to station loads (no body armor).

Various fitness measures have been found to relate to occupational load carriage ability [4,16]. For example, Robinson, et al. [17] conducted a study of specialist tactical response police carrying 20 kg of load as fast as possible over a 5 km distance at three different time intervals (several months apart). In their study it was found that strength measures of repetition maximum (RM) (bench press, squat, and pull-up), lower-body power (vertical jump (VJ)), and aerobic (multistage fitness, progressive shuttle run, or ‘beep’, test) performances were significantly correlated with all three load carriage performance events. As such, it is not surprising that research has found the combination of resistance and aerobic training as best associated with improvements in load carriage ability [18,19], and as such, they form the recommendations for physically conditioning tactical personnel to carry loads [20,21]. However, the majority of this research has focused on time to complete a distance march with loads above those required of general duties police, as opposed to shorter distances with the lighter loads utilized by general duties police. 

Female police officers may have to pursue offenders on foot, while wearing occupational loads that are relatively heavier than those carried by male officers. These occupational loads reduce mobility and are associated with measures of fitness. Therefore, understanding the relationships between fitness measures and load carriage during a change of direction speed (CODS) task may help inform physical conditioning requirements to optimize their ability to pursue offenders on foot whilst wearing occupational loads. On this basis, the aims of this research were to investigate the impacts of the occupational loads carried by female general duties police officers on a short explosive CODS task and determine which measures of fitness were related to this occupational load carriage requirement.

## 2. Materials and Methods 

Retrospective data were provided for 27 healthy female police officers (age = 32.19 ± 5.09 y, height = 162.78 ± 5.01 cm, and mass = 71.31 ± 13.42 kg) from the Abu Dhabi Police and included fitness measures of lower-body power (SLJ), upper-body and trunk muscle endurance (push-up (PU), sit-up (SU), aerobic power (estimated VO_2max_), and CODS (Illinois agility test). The female officers who applied to take part in sports activities as part of the competition and teams section of the Abu Dhabi Police were recruited for this study. Only the participants with no history of injuries or cardiovascular illness underwent testing procedures. Research was carried out in accordance with the conditions of the Declaration of Helsinki, recommendations guiding physicians in biomedical research involving human subjects [22], and with the ethical approval (number 484-2) from the ethical board of the Faculty of Sport and Physical Education, University of Belgrade.

### 2.1. Procedures

The explosive power of leg extensors was assessed by the SLJ test. Markovic [23] reported a high intratrial reliability for this test (Intraclass Correlation Coefficient = 0.95). The participants were instructed to jump as far as possible by performing a standing jump from a standard standing position. The distance from the starting line to the landing point at the heel contact was used for further analysis. The precision of the measurement was to the nearest 1 cm. 

**Figure 1:**
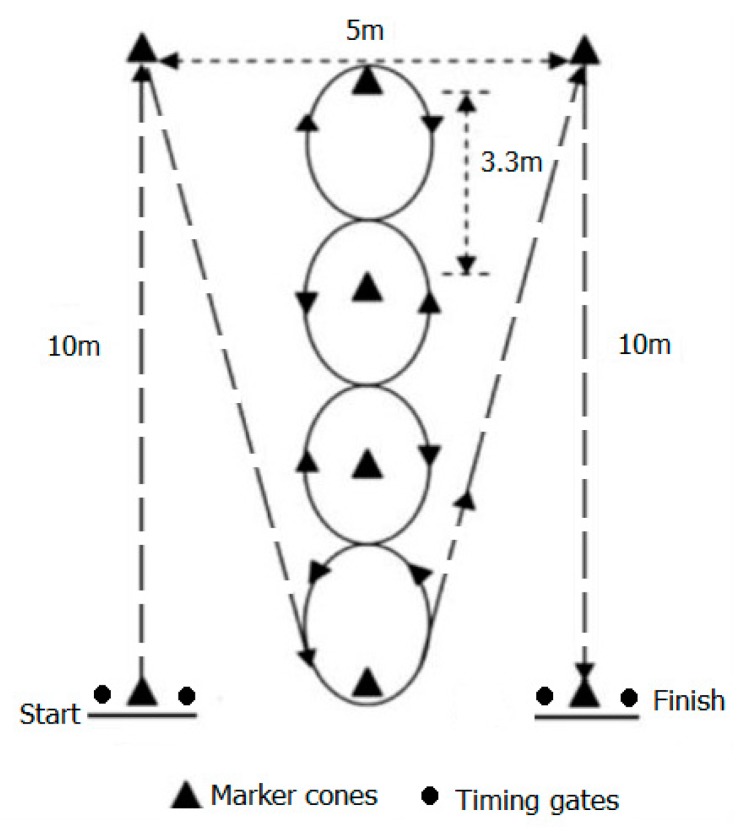
Schematic of the Illinois agility test.

The Illinois agility test was used as the measure of CODS [24]. Hachana [25] reported a high intratrial reliability of this test (ICC = 0.96). In addition to a standard Illinois agility test, the participants in this research also wore a 10 kg vest (Illinois agility loaded). A Star Fitness™ (Tortola, British Virgin Islands) adjustable weighted vest was firmly tightened to the upper body with two side straps that overlapped about the waistline in the front. The weight of the west was equally distributed at the front and back of the trunk. This load alone, without a sidearm or accoutrements, provided the 10 kg load. Following a 10 min respite from the SLJ, Illinois agility tests were performed with outcomes of both tests recorded using electronic timing gates (Fitro Light Gates, Fitronic, Bratislava, Slovakia). Measurement precision was to the nearest 0.01 s. The test course was used as previously reported in literature [24,26]. Two cones were used to mark the turning points, while four center cones were placed down the middle of the square grid and spaced 3.3 m apart for the weaving component (see Figure 1). The participants began the test lying prone on the floor behind the starting line. The timing gate was positioned 1 m above the starting line so the participants triggered the signal when they had already commenced the push up off the ground and started to move forward. On command, the participants stood and ran forward to the first turning cone in a straight line as fast as possible. The participants were required to turn around the first turning cone and moved back to the first center cone, where they weaved up and back around the four center cones. The participants then ran to the second turning cone. After turning around this cone, the participants were required to run in a straight line past the finish line. Following a slow then slightly faster (but submaximal) completion of the course as a warmup, participants were instructed to complete the test as quickly as possible. Participants were familiar with the course and repeated the course twice, firstly without load and then, after a short period of respite of 10 min, with load.

The 1 min PU and SU tests were conducted 10 min after the Illinois agility test following the procedures previously described in the literature [27], with the exception of the PU, which was completed on the knees rather than the toes. In short, each participant was positioned so that only a maximum of four points contacted the ground (knees and hands) while the body was straight from heels to head. The participants were advised that the between-hand width should be approximately one palm wider than their shoulder width. The starting position had the arms fully extended with one PU counted when the elbow joint bent to at least 90 degrees and then extended back to the starting position. After the PU test, participants rested for 15 min before completing the SU test. Participants started the test from a laying position with hands crossed at chest height, palms on the opposing shoulders, and knees bent at an angle of 90°. The feet were placed flat on the ground and secured by the tester. One repetition was counted when the participant completed an SU by raising the upper body and touching the knees with elbows. Hips had to maintain contact with the ground, and hands had to remain on the chest during the full range of movement throughout the test. The only permissible resting position was the ‘start’ position. Every SU that did not meet these standards were not counted. For both the PU and SU, participants were required to complete as many repetitions as possible in the time or until volition fatigue. Results were measured in single repetitions.

Female police officer VO_2max_ values were estimated using an incremental, multistage 20 m shuttle-run test on an indoor rubber matt, according to previously reported procedures [28]. This test was conducted 10 min after the SU test. Shuttle run levels were controlled using the mobile app beep test, ‘police military multistage assessment’ connected to a loudspeaker so each change of the level was clearly and loudly announced. After the test was finished, results were written down in forms of levels and shuttles attained by each participant. To further estimate VO_2max_, results were typed into a Microsoft Excel file (Microsoft Corporation^TM^, Redmond, Washington, DC, USA) and calculated using the formula developed by Ramsbottom et al. [29], which was based on age and completed number of levels and shuttles.

### 2.2. Statistics

Data were transferred from a Microsoft Excel spreadsheet, on which the data were recorded, into Statistical Package for the Social Sciences (SPSS version 25, Chicago, IL, USA) for analysis. Following descriptive analysis and tests for normality and homogeneity, paired sample *t*-tests were used to determine differences between loaded and unloaded conditions. Pearson’s product correlations were performed to investigate relationships between load conditions and fitness variables. A regression analysis was performed with all significantly correlated variables to determine how much of the CODS task could be attributed to these variables. The regressions were conducted both including and excluding the opposing CODS task (i.e., loaded/unloaded CODS task). Alpha levels were set at 0.05 a priori.

## 3. Results

Descriptive data are provided in Table 1. The results of the paired sample *t*-test indicated that female officers were significantly slower (t(26) = −6.001, *p* < 0.001) when performing the CODS task while wearing occupational loads. 

All fitness measures were significantly correlated with both the unloaded and loaded Illinois agility run, with the strength of the correlations [30] ranging from moderate (estimated VO_2max_) to strong (SU) (Table 2). In all cases, the strength of the correlations between the fitness measures and the Illinois agility run increased when load was added. As would be expected, the unloaded Illinois agility test was very strongly correlated with the Illinois agility when loaded (*r* = 0.956, *p* < 0.001).

When all measures that correlated to the unloaded CODS were entered into the regression, the variables equated to 92% of the variance, dropping to 61% of the variance when the loaded CODS was removed from the regression. Subsequently, when all measures that correlated to the loaded CODS were entered into the regression, the variables equated to 93% of the variance, dropping to 67% of the variance when the unloaded CODS was removed from the regression.

## 4. Discussion

The aims of this research were to investigate the impacts of the occupational loads carried by female general duties police officers of the Abu Dhabi Police on a CODS task and determine which measures of fitness were related to this occupational load carriage requirement. The study found that when female police officers wore a 10 kg duty load their CODS was significantly slower. Furthermore, while moderate-to-strong significant relationships were found between all fitness measures and CODS, the strength of these relationships increased in the loaded condition. 

The results of this study suggested that lower-body power, upper-body and trunk endurance, and aerobic fitness are associated with police officer CODS, especially when the officer was wearing occupational load. Previous research has found that measures of fitness are associated with mobility-styled activities in law enforcement personnel [31]. Lockie et al. [31] found that SU (*r* = −0.208), pull-ups (*r* = −0.272) and 2.4 km run (*r* = 0.253) performances were all associated with a 99-yard (90.53 m) obstacle course run, which was designed to simulate a foot pursuit and required police recruits to step over simulated curbs and high obstacles over the course. Similarly, Dawes et al. [32] found a moderate to strong relationship between VJ and sprint performance (5–20 m) in specialist police officers—findings that were supported by Marques et al. [33,34] and Wisløff, et al. [35]. Thus, VJ was strongly associated with short distance sprints, which was a performance measure known to be reduced by the wearing of body armor in police [36,37].

In a military population, soldiers wearing and carrying a total load of approximately 42 kg completed an anaerobic based task, which included a 27 m zigzag run, and they were significantly slower when carrying their additional loads [38]. Of note, and in support of aforementioned research, the initial 5 m start, in particular, was found to be significantly slower when the soldiers were loaded [38]. However, the zigzag component (while slower) was not shown to be significantly different between load conditions. Considering this, the study found significant, strong correlations between overall course performance times in both the unloaded and loaded conditions with lower body strength (1 RM squat), lower-body power (peak power), and upper-body strength (1 RM bench press). PU, SU, and a 2 mi run were not significantly correlated with performance on the course (in either load condition) [38]. Of note, the study found PUs were significantly correlated with time to rise from the prone position to begin the initial 5 m sprint of the course. This coincided with other research, which suggested that PU ability was related to other military-styled tasks that required the load carrier to rise from a prone position [12,39]. This also coincided with this current study, whereby female police officers were required to rise from a prone position to commence the initial forward sprint.

The CODS time to completion was between 23.17 ± 2.75 s (unloaded) to 24.14 ± 2.78 s (loaded) and was an anaerobic task [40]. As such, correlation with aerobic performance measures was not expected. For example, in the aforementioned research by Mala et al. [38], there was no correlation between two-mile run times and performance on a short, explosive anaerobic task (from 25.4 ± 1.8 s unloaded to 38.7 ± 4.8 s when loaded). However, Lockie et al. [31] did likewise find that 2.4 km run times were associated with several work sample test battery tasks ranging from a solid wall fence climb (7.75 ± 1.37 s) to a longer 500-yard run (89.20 ± 7.99 s). A potential reason for the findings of this study may lie in the fact that the officers may have been more likely to be more physically active (as the testing occurred at the end of a 22 week academy) and would have completed more physical activity (and hence be more fit in general).

The findings of this study, whereby measures of fitness were correlated to CODS (more so when the officers were loaded), and whereby unloaded CODS was very strongly correlated to loaded CODS, provide some guidance for the conditioning of female police officers who are required to wear occupational loads and pursue a suspect on foot. Poor CODS performance can be improved by increasing general fitness, more specifically PU, SU, and SLJ ability. Likewise, unloaded CODS ability can be used to inform loaded CODS potential and allow for this progression when a suitable unloaded CODS has been achieved.

### Limitations

Key limitations to this study were the inability to change assessment order or to randomize the unloaded and loaded conditions. While these restrictions are common in retrospective cohort studies, familiarization of the course is expected to minimize any potential learning effect induced by the assessment order. Furthermore, long breaks of over 10 min between each condition, a period exceeding the recommended recovery period of power-based activities [41], should mitigate any fatigue concerns. It should also be noted that female police officer participants were from a group which may have been more physically active, and as such, the influence of fitness measures on CODS performance may not be reflective of less physically active female police officers.

## 5. Conclusions

While unloaded agility performance was strongly associated with loaded performance, female police officer CODS was reduced when officers carried occupational loads. All fitness measures were correlated with unloaded CODS performance, with this relationship increasing when occupational loads were worn. As such, upper-body and trunk endurance, lower-body power, and aerobic fitness become increasingly important when preparing female police officers to carry occupational loads. Furthermore, unloaded CODS performance (as measured by the Illinois agility test) can be used to gauge readiness to perform loaded CODS.

## Figures and Tables

**Table 1:** Demographic and fitness measure results.

	Mean ± SD (Range)
Age (y)	32.19 ± 5.09 (22.00–42.00)
Height (cm)	162.78 ± 5.01 (155.00–173.00)
Mass (kg)	71.31 ± 13.42 (50.50–109.50)
Body mass index (kg/m^2^)	26.86 ± 4.57 (20.80–36.60)
Push-Ups (repetitions)	24.04 ± 11.77 (7.00–49.00)
Sit-Ups (repetitions)	28.48 ± 10.79 (13.00–53.00)
Standing Long Jump (cm)	166.00 ± 25.81 (116.00–210.00)
Estimated VO_2max_ (mL/kg/min)	24.32 ± 4.32 (19.55–35.06)
Illinois agility (s)	23.17 ± 2.75 (18.58–28.21)
Illinois agility loaded with 10 kg (s)	24.14 ± 2.78 (18.96–29.86)

SD: Standard Deviation.

**Table 2:** Correlations between fitness measures and Illinois agility test in both the unloaded and loaded conditions.

	SLJ	SU	PU	Estimated VO_2max_	Illinois Agility	Illinois Agility Loaded (10 kg)
Illinois agility	−0.649 **	−0.673 **	−0.605 **	−0.448 *	1	0.956 **
Illinois agility loaded (10 kg)	−0.686 **	−0.707 **	−0.624 **	−0.514 **	0.956 **	1

Correlation is significant at: * *p* < 0.05, ** *p* < 0.01. SLJ = standing long jump; SU = sit-up; and PU = push-up.
